# Mortality and comorbidities in patients with bronchiectasis over a 3-year follow-up

**DOI:** 10.1097/MD.0000000000032537

**Published:** 2022-12-30

**Authors:** Simone Paulo Mateus, Marcelo Ribeiro-Alves, Raquel Esteves Brandão Salles, Walter Costa, Claudia Henrique da Costa, Agnaldo José Lopes, Thiago Prudente Bártholo, Thiago Thomaz Mafort, Bernardo Rangel Tura, Rogério Rufino

**Affiliations:** a Department of Chest Diseases, ^-^Rio de Janeiro State University, Pulmonology Service, Rio de Janeiro, Brazil; b Fundação Oswaldo Cruz, Rio de Janeiro, Brazil; c National Institute of Cardiology, Laranjeiras, Brazil.

**Keywords:** bronchiectasis, exacerbation, hospitalization, mortality, quality of life, spirometry

## Abstract

To identify the risk factors associated with all-cause mortality in patients with noncystic fibrosis bronchiectasis (NCFB). This prospective cohort study included 120 adult patients with NCFB, who were regularly treated at a specialized outpatient clinic of a university hospital between January 2017 and June 2020. All patients were diagnosed using high-resolution computed tomography. Demographic and clinical data, pulmonary function tests, and the Euro-quality-of-life 5-domain 3-level questionnaire were analyzed. The factors associated with death were determined using the Cox proportional hazards model. The all-cause mortality rate at 41 months was 10.8%. Adjusted multivariate analysis showed that the main contributing predictors for mortality were female sex, smoking, diabetes, chronic obstructive pulmonary disease, emergency visits, use of antibiotics due to exacerbation, secretion color change, exacerbation, predicted forced expiratory volume in 1 second, predicted forced vital capacity, lack of respiratory physiotherapy, absence of vaccination against pneumococci, and mobility domain. Multiple factors contribute to unfavorable outcomes in patients with NCFB, and early recognition of these factors may improve care management.

## 1. Introduction

Noncystic fibrosis bronchiectasis (NCFB) is a chronic, irreversible respiratory disease with a clinical course that oscillates between periods of exacerbation and spontaneously decreased symptoms. Bronchiectasis can affect people at any stage of life; however, it has the highest incidence among older people.^[1–3]^ In recent decades, an increasing number of cases of bronchiectasis have been reported, mainly due to the introduction of high-resolution computed tomography (HRCT)^[4,5]^ for its diagnosis. HRCT can also estimate the extent of the disease and monitor its progression.^[4,5]^

The etiologies vary geographically^[1,3]^ and may be associated with congenital or acquired conditions. There are many cases with undetermined etiologies, despite performing all tests recommended for etiological investigation, identified as idiopathic bronchiectasis.^[4]^ Various comorbidities are often associated with bronchiectasis.^[5,6]^ The coexistence of other diseases can be a risk factor for mortality in patients with bronchiectasis.^[6,7]^ Some predictors have been correlated with higher mortality in patients with NCFB; however, these predictors have not been well determined in Brazilian patients.^[8]^ A better understanding of risk factors would optimize care and establish more efficient therapeutic approaches. Therefore, this study aimed to identify risk factors for mortality in patients with NCFB during outpatient follow-up at a tertiary institution.

## 2. Methods

### 2.1. Patients and study design

This prospective, single-center cohort study enrolled patients with NCFB who were followed-up for 3 years from January 2017 to June 2020. The study was approved by the Research Ethics Committee of Pedro Ernesto University Hospital (no. 1823,665). All the participants signed an informed consent form.

The patients were diagnosed with bronchiectasis confirmed by chest HRCT, which was performed by 2 radiologists and 2 pulmonologists. Demographic and clinical data, pulmonary function tests, and the Euro-quality-of-life (QoL) 5-domain 3-level (EQ-5D-3L) questionnaire were collected and administered during the initial interview.^[9]^ The following variables were included: sex, age, weight, height, dyspnea scale [modified Medical Research Council], number of emergency room visits, antibiotic usage and purpose, secretion volume and appearance, presence of hemoptysis, number of exacerbations, hospitalization in the previous year, etiology, number of lung lobes affected on chest HRCT, use of oxygen therapy, infectious agents in culture, smoking, comorbidities, history of pulmonary tuberculosis, previous surgery/thoracic resection, vaccination for influenza and pneumococcus, regular respiratory physiotherapy, and history of recurrent pneumonia (Additional file). Patients with bronchiectasis were classified into 2 groups: group of survivors (GS) and group of patients who died (GD) after a 3-year follow-up period.

### 2.2. Variables

Bronchiectasis cases under etiological investigation were defined as those with undetermined etiology. Exacerbation was defined as patient care in an unscheduled or emergency outpatient unit, with or without the need for antibiotic therapy, and the association of at least 3 of 4 clinical signs (increased intensity of dyspnea, increased daily sputum volume, secretion color change, or fever).^[10,11]^ Chronic colonization was defined as the isolation of the same pathogen in 2 or more positive cultures within 1 year, with a minimum interval of 3 months between samples.^[4]^ Sputum was classified as mucoid (light), mucopurulent (pale yellow or pale green), or purulent (dark yellow or dark green).^[12]^ Survival time was measured from the date of clinical diagnosis, or in case of its absence, the date of the first consultation at the medical specialty clinic until death or termination of the study, whichever occurred first. The follow-up method was based on routine medical consultation or telephone contact with the patient or family member (Table 1) when the interval was longer than 3 months.

**Table 1:** Cox proportional hazard analysis of factors associated with mortality in patients with bronchiectasis

	aHR *	95% confidence interval	*P* value
Demographic data			
Female	8.14	2.39–27.78	.001
BMI, kg/m² (< 24.9)	0.12	0.04–0.36	.0002
Etiology (postinfection nontuberculosis)	0.13	0.04–0.52	.004
Comorbidities			
Diabetes	3.92	1.09–14.10	.037
COPD	4.03	1.28–12.74	.018
Current smoker	22.46	2.03–248.74	.011
Worsening of symptoms, previous year			
Emergency visit, (twice)	36.77	4.10–330.14	.001
Exacerbation	3.78	1.05–13.65	.042
Sputum color change	8.19	2.45–27.38	.001
Use of antibiotic due to exacerbation	11.17	2.25–55.48	.003
Spirometric data			
FEV_1_ % predicted (< 53.5)	6.51	1.60–26.46	.009
FVC % predicted (< 66.5)	36.17	5.72–228.59	.0001
Quality of life, EQ-5D-3L			
Mobility	5.57	1.46–21.21	.012
Anxiety/depression	2.91	0.82–10.39	.100
Valuation (< 0.65)	0.57	0.16–2.03	.382
VAS (<70)	4.41	1.33–14.59	.015
Prophylactic measures			
Absence of respiratory physiotherapy	41.32	4.97–343.68	.001
Influenza vaccination	0.09	0.02–0.36	.001
Absence of pneumococcal vaccination	7.00	2.90–24.48	.002

aHR* = adjusted hazard ratio, BMI= body mass index; COPD = chronic obstructive pulmonary disease; FEV_1_ = forced expiratory volume in 1 second; FVC = forced vital capacity; VAS = Visual Analogue Scale.

### 2.3. Statistical analyses

The Mann–Whitney *U* test was used to compare baseline demographic and clinical data for continuous numerical variables, and chi-square tests were used for categorical nominal variables to evaluate frequency independence between these variables and death. To estimate the hazard of progression to death, person-years (pY) at risk were calculated based on the number of follow-up years with bronchiectasis for each patient. Live patients were censored at the end of the follow-up period. The effects of various risk factors on death were assessed using adjusted hazard ratios and their corresponding 95% confidence intervals, which were estimated using Cox proportional hazards multiple regression models. In addition to disease types, any clinical or phenotypic features at least suggestively associated with the outcome were introduced as confounders (*P* ≤ .1) to eliminate any possible bias introduced by convenience sampling. Whenever needed, continuous numerical variables were categorized using the round integer number closest to either the median or the percentiles 0.33 and 0.66, respectively. In addition, the prevalence rates and 95% confidence interval of patients with bronchiectasis were estimated according to asymptotic standard errors calculated from a gamma distribution. Two-tailed levels of significance ≤ 0.01, 0.05, and 0.1 were considered “highly significant,” “significant,” and “suggestive,” respectively. All statistical analyses were performed using R software version 3.6.4.

## 3. Results

### 3.1. Baseline characteristics

A total of 121 patients who were regularly monitored at the bronchiectasis outpatient clinic of the Department of Pulmonology of Pedro Ernesto University Hospital, Rio de Janeiro, RJ, Brazil, were eligible for the study. One participant withdrew consent and was excluded. The analyses were performed for 120 patients. Thirteen deaths (10.8%) occurred during the study. The median survival time (interquartile range, IQR) was 4.19 (2.19–10.87) years in the GD group. The baseline and clinical characteristics of the patients included in the study are summarized in Table 2. The analysis identified that patients in the GD group were older than those in the GS group, with median ages of 71 (IQR = 23) and 59 years (IQR = 18.5), respectively. Regarding the sex distribution, there was a predominance of women in both groups (72% in the GS group and 53.8% in the GD group). Most patients in the GS group had a normal body mass index (BMI), which was significantly lower in the GD group (*P* = .014). There were no statistically significant differences between the 2 groups in terms of dyspnea, smoking, hemoptysis, emergency room visits, exacerbation, hospitalization, oral antibiotic therapy, and vaccination. In the GD group, dyspnea (modified Medical Research Council scale) was reported as grade 0 (7.7%), grade 1 (46.2%), grade 2 (38.5%), and grade 3 (7.7%). Dyspnea in the GS group was reported as grade 0 (15%), grade 1 (42.1%), grade 2 (20.6%), or grade 3 (22.4%). Both groups had a higher incidence of bronchiectasis related to pulmonary tuberculosis sequelae (38.5% in GS and 56.1% in GD). Pulmonary involvement in 2 or more lobes was identified by chest HRCT in all (100%) patients in the GD group and 86 (80.4%) patients in the GS group, with no significant difference between the 2 groups. Of the 13 patients who died, 12 (92.3%) presented with hypersecretion, and 9 (69.2%) had a history of multiple comorbidities. The most prevalent coexisting disease in the GD group was systemic arterial hypertension in 61.5% of patients, followed by chronic obstructive pulmonary disease (COPD) (38.5%) and diabetes mellitus (30.8%). Additionally, these patients had a higher frequency of recurrent pneumonia (69.2%) and rhinosinusitis (69.2%). Four (30.8%) patients had visited the emergency care unit at least once in the previous year because of exacerbation. However, none of the patients were hospitalized. In the GS group, 3 patients (2.5%) required hospital admission because of severe exacerbation. Hemoptysis was reported by the patients in both groups (GS, 11.2%; GS, 7.7%). Furthermore, 32.7% of the patients in the GS group showed no secretion, 25.2% had mucoid secretions, 35.8% had purulent secretions, and 6.5% had mucopurulent sputum. In the GD group, 7.7% of the patients showed no secretion, most of them (53.8%) had mucoid secretion, and 38.5% had purulent sputum.

**Table 2:** General characteristics of the study population with nonfibrocystic bronchiectasis

Variables	Overall cohort	Alive	Died	*P* value
Number of patients, n	120	107	13	
Female, n (%)	84 (70.0)	77 (72.0)	7 (53.8)	.305
Age, year, median (IQR)	59.50 (19.25)	59.0 (18.50)	71.0 (23.00)	.106
BMI, kg/m², median (IQR)	23.64 (6.27)	24.02 (6.07)	20.66 (6.06)	.014
Comorbidity, n (%)				
Without comorbidities	24 (20.0)	21 (19.6)	3 (23.1)	.188
With comorbidities	35 (29.2)	34 (31.8)	1 (7.7)	
≥ 2 comorbidities	61 (50.8)	52 (48.6)	9 (69.2)	
Systemic arterial hypertension, n (%)	57 (47.5)	49 (45.8)	8 (61.5)	.436
Cardiovascular disease, n (%)	11 (9.2)	11 (10.3)	0 (0.0)	.481
Diabetes mellitus, n (%)	19 (15.8)	15 (14.0)	4 (30.8)	.246
GERD, n (%)	11 (9.2)	11 (10.3)	0 (0.0)	.481
Cardiovascular disease, n (%)	14 (11.7)	12 (11.2)	2 (15.4)	1
Neoplastic disease, n (%)	9 (7.5)	7 (6.5)	2 (15.4)	.558
Hypothyroidism, n (%)	4 (3.3)	2 (1.9)	2 (15.4)	.081
COPD, n (%)	33 (27.5)	28 (26.2)	5 (38.5)	.543
Asthma, n (%)	24 (20.0)	23 (21.5)	1 (7.7)	.419
Previous tuberculosis, n (%)	68 (56.7)	62 (57.9)	6 (46.2)	.607
Rhinosinusitis, n (%)	68 (56.7)	59 (55.1)	9 (69.2)	.502
Recurrent pneumonia, n (%)	65 (54.2)	56 (52.3)	9 (69.2)	.390
Smoking status, n (%)				
Never smoker	94 (78.3)	85 (79.4)	9 (69.2)	.826
Active smoker	6 (5.0)	5 (4.7)	1 (7.7)	
Ex-smoker	15 (12.5)	13 (12.1)	2 (15.4)	
Passive smoker	5 (4.2)	4 (3.7)	1 (7.7)	
Etiology, n (%)				
Idiopathic	15 (12.5)	14 (13.1)	1 (7.7)	.239
Postinfection tuberculosis	65 (54.2)	60 (56.1)	5 (38.5)	
Postinfection nontuberculosis	25 (20.8)	22 (20.6)	3 (23.1)	
Undetermined*	12 (10.0)	9 (8.4)	3 (23.1)	
Kartagener syndrome	2 (1.7)	1 (0.9)	1 (7.7)	
Primary immunodeficiency	1 (0.8)	1 (0.9)	0 (0.0)	
Affected lobes, n (%)				
One lobe	21 (17.5)	21 (19.6)	0 (0.0)	.170
≥ 2 lobes	99 (82.5)	86 (80.4)	13 (100.0)	
Colonization *Pseudomonas aeruginosa,* n (%)	22 (18.3)	19 (17.8)	3 (23.1)	.929
Colonization others microorganisms†, n (%)	16 (13.3)	15 (14.0)	1 (7.7)	.840
Baseline PFT, n	100	89	11	
FEV_1_ (% predicted), median (IQR)	53.35 (35.17)	56.00 (36.90)	34.40 (17.95)	.023
FVC (% predicted), median (IQR)	66.40 (29.38)	69.00 (29.20)	50.90 (16.45)	.025
FEV1/FVC ratio (%), median (IQR)	79.55 (24.92)	82.50 (23.50)	66.20 (17.20)	.024

BMI = body mass index; COPD = chronic obstructive pulmonary disease; FEV_1_ = forced expiratory volume in 1 second; FVC = forced vital capacity; GERD = gastroesophageal reflux disease; IQR = interquartile range; n = number of patients (percentage); PFT = pulmonary functional test.

* Undetermined: cases in etiological investigation.

† *Staphylococcus aureus, Haemophilus spp.*, *Aspergillus flavus* and nontuberculous *mycobacteriosis*. Obs. Analyzed with the Mann–Whitney *U* test for continuous variables and the chi-square test for categorical variables.

*Pseudomonas aeruginosa* colonization was observed in sputum samples from both groups (GS, 17.8%; GS, 23.1%). Half of the patients (50%) received oral antibiotic therapy in the previous year. In the GS group, 26 (21.7%) patients used it prophylactically, 26 (21.7%) used it because of exacerbation, and 8 (6.7%) used both therapies concomitantly. In addition, 4 patients (30.8%) used prophylactic antibiotic therapy, 2 (15.4%) used it because of exacerbation, and 2 (15.4%) used both therapies concomitantly.

In addition, more than half of the patients in both groups received influenza vaccination (GS, 81.3%; GS, 76.9%), with similar pneumococcal immunization rates in both groups (35.5% for GS and 38.5% for GD). Less than 8% of the study population were enrolled in a routine respiratory physiotherapy program (GS, 7.5%; GS, 7.7%). The comparison of spirometry results between the 2 groups showed statistically significant differences in forced expiratory volume in 1 second (FEV_1_) (*P *= .023), forced vital capacity (FVC) (*P* = .025), and FEV_1_/FVC ratio (*P *= .024). QoL was assessed using the EQ-5D-3L questionnaire, which showed that most patients in both groups had moderate problems in 4 of the 5 dimensions: mobility (61.7% in GS and 76.9% in GD), difficulties in performing their usual activities (72.9% in GS and 69.2% in GD), pain/malaise (62.6% in GS and 76.9% in GD), and anxiety/depression (50.5% in GS and 53.8% in GD). Both groups showed no statistically significant differences in QoL parameters.

### 3.2. Mortality and causes of death

Overall, 13 (10.8%) patients died of various causes during the study period. No deaths occurred during the first year of the follow-up. However, 8 patients died in the second year and 5 in the third year (6.6% and 4.2%, respectively). Deaths related to the circulatory system accounted for 30.8% of all deaths, followed by infectious and parasitic diseases (23.1%), neoplasms (15.4%), digestive system diseases (15.4%), respiratory system diseases (7.7%), and external morbidities and mortality (7.7%). According to the International Classification of Diseases, the causes of death during the study period were acute myocardial infarction, cerebrovascular diseases, liver fibrosis and cirrhosis, digestive hemorrhage, septicemia, human immunodeficiency virus diseases, COPD, and laryngeal and breast cancer.

### 3.3. Potential risk factors for mortality

Cox proportional hazards analysis results after adjusting for age, BMI, hypothyroidism, diabetes, and secretion appearance are shown in Table 1. Variables that were significantly associated with an increased risk of death were female sex, active smoking, diabetes, COPD, emergency room visits, use of oral antibiotics due to exacerbation, secretion color change, exacerbation, FEV_1_, FVC, lack of respiratory physiotherapy, lack of pneumococcal vaccination, mobility domain (EQ-5D-3L), and visual analog scale scores. However, a BMI within the normal range, postinfectious nontuberculosis etiology, and influenza vaccine had a protective effect on mortality.

## 4. Discussion

This study showed that sex, smoking, diabetes, COPD, emergency room visits, use of oral antibiotics due to exacerbation, sputum color change, exacerbation, spirometric indicators, lack of respiratory physiotherapy, absence of vaccination against pneumococci, limited mobility (EQ-5D-3L), and self-rated health (visual analog scale) correlated with the risk of mortality in patients with NCFB. Other factors, such as normal BMI, nontuberculosis postinfectious etiology, and regular influenza vaccination, played a protective role. This study also reported a high all-cause mortality rate of 10.8% after a mean follow-up period of 40 months. Cardiovascular disease accounted for 30.8% of all deaths in this cohort, with acute myocardial infarction as the main cause. There was a higher percentage of deaths from nonrespiratory causes, which may be explained by the prevalence of multiple comorbidities.

The mortality rate of patients with bronchiectasis has increased over the past decade. Despite this huge problem, few studies have focused on this issue in these patients. The mortality rate observed in the present study was similar to that reported in previous studies. Goeminne et al^[13]^ reported a mortality rate of 10.6% among 539 patients in a 41-month retrospective study. One study with 91 patients found a survival rate of 91% over a 4-year follow-up, 83.5% over an 8-year follow-up, and 68.3% after 12.3 years.^[14]^ Onem et al^[15]^ reported a mortality rate of 16.3% in 98 outpatients in a 4-year prospective study. A large study with an Asian population including 18,134 patients with bronchiectasis reported that 9.2% died over a mean follow-up period of 5.8 years.^[16]^

Hypertension, rhinosinusitis, recurrent pneumonia, COPD, asthma, and diabetes mellitus were the main comorbidities identified in the present cohort. The presence of comorbidities has been identified as a risk factor for increased mortality in patients with NCFB^[6,17]^; however, it was not considered a predictor of increased death rate in the present study. A previous study showed that comorbidities increase the mortality rate of patients with bronchiectasis.^[14]^

Bronchiectasis has numerous causes, although it has often been reported to coexist with COPD. COPD is also one of the factors associated with a higher risk of death. Some studies have shown that the association between bronchiectasis and COPD increases the risk of mortality.^[6,18]^ Post-TB sequelae are the main conditions associated with the development of bronchiectasis; however, this may be due to the high incidence of pulmonary tuberculosis infection in Brazil. There was no association between post-TB infection and a poor prognosis. Corroborating previous studies, countries with a high incidence of *Mycobacterium tuberculosis* infection commonly have high rates of postinfectious tuberculosis bronchiectasis cases.^[19–22]^

Exacerbation was also identified as a risk factor for death in patients with bronchiectasis in the present cohort. The clinical course of bronchiectasis can be marked by exacerbations ranging from the onset of slightly increased respiratory symptoms to respiratory failure, which is the most severe presentation.^[10]^ Increased sputum volume, purulence, cough exacerbation, dyspnea, and systemic symptoms are frequent exacerbations.^[10]^ Chang et al^[23]^ reported that exacerbations worsen QoL, reduce lung function,^[8]^ increase hospitalization, and result in a long-term respiratory decline.

FEV_1_ and FVC were correlated with worse prognosis in patients with NCFB. Airflow limitation has been associated with an increased risk of mortality.^[16,24]^ Spirometric data can help to assess disease progression and severity. Patients with NCFB often present with obstructive disorder.^[7,15]^

This study showed compromised QoL in the study cohort using the EQ-5D-3L questionnaire.^[8]^ This finding corroborates the cross-sectional study conducted in Korea with 19,851 participants with and without bronchiectasis, which reported worse QoL and mobility difficulties in patients with bronchiectasis.^[20]^ The present study showed that mobility domain is associated with the risk of death.

Lack of pneumococcal vaccination is another risk factor contributing to mortality. Approximately 65% of the patients had no pneumococcal vaccination. Most patients received regular influenza vaccination. A protective relationship between regular influenza vaccination and mortality was observed. Previous studies have recommended the use of pneumococcal vaccines^[25,26]^ in patients with chronic lung diseases and reported that they can reduce the risk of complications and death.^[4,27]^ Menéndez et al,^[28]^ patients with bronchiectasis who were immunized with the pneumococcal vaccine had reduced hospitalizations due to exacerbations. Preventive measures, including vaccination and regular medical appointments, are associated with better survival in patients with NCFB.^[15]^ Patients with incomplete immunization are referred to the Immunobiological Reference Center. Less than 10% of the patients were included in a respiratory rehabilitation program, possibly because of difficult access to the health system.

This study had some limitations. First, the study population was recruited from a single center and consisted of a limited number of participants. Second, the time to survival follow-up may be considered short, and many patients did not undergo rehabilitation or preventive vaccination, despite being instructed to do so.

In conclusion, this study identified potential factors associated with the risk of mortality in patients with bronchiectasis and highlighted that some of these factors are modifiable and can therefore be addressed using a preventive approach.

## Acknowledgments

We would like to thank the Pulmonology Service, Department of Chest Diseases, Medical Sciences Faculty, Rio de Janeiro State University, Brazil.

## Author contributions

**Conception and design:** Simone Paulo Mateus, Bernardo Rangel Tura, Marcelo Ribeiro-Alves, Rogerio Rufino.

**Data acquisition:** Simone Paulo Mateus, Raquel Esteves Brandão Salles, Walter Costa, Claudia Henrique da Costa, Agnaldo José Lopes, Thiago Prudente Bártholo, Thiago Thomaz Mafort.

**Data analysis and interpretation:** Simone Paulo Mateus, Marcelo Ribeiro-Alves, Rogerio Rufino.

**Supervision:** Rogerio Rogerio.

**Conceptualization:** Simone Paulo Mateus, Bernardo Rangel Tura, Rogerio Rogerio.

**Data curation:** Simone Paulo Mateus, Rogerio Rogerio.

**Formal analysis:** Marcelo Ribeiro-Alves, Rogerio Rogerio.

**Funding acquisition:** Rogerio Rogerio.

**Investigation:** Raquel Esteves Brandão Salles, Claudia Henrique da Costa, Agnaldo José Lopes, Thiago Prudente Bártholo, Thiago Thomaz Mafort, Rogerio Rogerio.

**Methodology:** Simone Paulo Mateus, Marcelo Ribeiro-Alves, Raquel Esteves Brandão Salles, Walter Costa, Claudia Henrique da Costa, Agnaldo José Lopes, Thiago Prudente Bártholo, Thiago Thomaz Mafort, Rogerio Rogerio.

**Project administration:** Rogerio Rogerio.

**Supervision:** Rogerio Rogerio.

**Validation:** Simone Paulo Mateus, Rogerio Rogerio.

**Visualization:** Rogerio Rogerio, Simone Paulo Mateus, Rogerio Rogerio.

**Writing—original draft:** Simone Paulo Mateus, Rogerio Rogerio.

**Writing—review and editing:** Simone Paulo Mateus, Rogerio Rogerio.
